# Everybody's Page

**Published:** 1889-06-22

**Authors:** 


### Everybody's Page.

Between five and six ounces of blood is forced into the arteries at each contraction of the heart, and as much is brought back to the heart by the veins. The amount of force exerted by the heart during twenty-four hours would, it has been calculated, raise a weight of 122 tons a foot from the ground.

The first effect of alcohol on the system is to accelerate the action of the heart, and raise the temperature of the body about one and a half degrees. It is this effect which makes it valuable in cases of fainting or collapse. The secondary effect is, however, to lower the temperature, which sometimes falls two or three degrees below the normal point of 98deg., and the warmth of the body cannot be restored as quickly as it is lost. For this reason drunkards are more likely to suffer from exposure to cold than temperate people, and the stupor of alcohol is apt to pass into the sleep of death.

The invention of omnibuses is due to the philosopher Pascal, who in February, 1667, obtained a "privilege"—what we should call a patent—for public carriages to travel through certain streets of Paris. They held eight passengers, who paid six sous each, and were very successful, although an Act of the Parliament of Paris forbade them being used by soldiers, lackeys, and other humble folks. Pascal died in 1667, and his useful invention did not long survive him. The omnibus reappeared in London about the beginning of the century, and was adopted in several French provincial towns before Paris accepted it again.

The *Globe* indulges in a doleful article on the establishment in New York of what are called "Dorothy chambers," that is, chambers and flats for women only. It looks upon the fact of women—young women—living unchaperoned and alone, as one of the signs of the times in America, and is thankful, solemnly thankful, that the idea of the "Dorothy chambers" will not be imported into this country. Here, when women have to leave home to earn their living, they put themselves, the *Globe* believes, under the care of some responsible head of a family. Not always, we think. The "Dorothy" fashion will not be imported into England, because it is there already. Has the *Globe* never heard of the Campden Houses, the Oakley Flats, the recently opened flats for women in Sloane-square, and still more recently finished ladies' chambers in Chenies-street? The intention in all these is that the occupants, who are for the most part ladies who work for themselves, may live in the complete independence which is almost necessary to success in any profession.

"Her 'prentice han' she tried on man, and then she made the lassies," says Burns, and every woman firmly believes that his theory of "Dame Nature's" methods is absolutely correct. But, say the sceptics, although women have, no doubt, finer senses than men—have some seven or seventeen mystic senses that a man doesn't share, and can't for the life of him understand—in one faculty, the sense of smell, she is infinitely inferior to him. Her "tip-tilted" nose—we thank thee, poet, for teaching us that word!—is insignificant compared with the manly aquiline of her kindred of the baser sex. Is this a victory for the sceptic? No, certainly not. The sense of smell is stronger and of more practical value in savage than in civilised races; in the lower animals than in man. A Red Indian will trace friend or foe by the scent, like a dog. Is the Red Indian, then, higher in the scale of humanity than the average Englishman? And if not, if the possession of a keen sense of smell proves no superiority, the lack of it in women can show no inferiority. You can't have it both ways, gentlemen.

Ambulances and surgical dressings are not confined to the human race. The warrior ants take the greatest care of their wounded, and Latrielle, the naturalist, records how, when he cut the antennæ of an ant, other ants came and covered the wounded part with a transparent fluid secreted in their mouths.

An interesting story is told of the late M. Cheareul's keen sense of smell. One day he suddenly turned to one of his pupils, saying "I am sorry you smoke. I thought you did not." "I don't as a rule," replied the pupil, "and it is now more than three hours since I smoked half a cigarette. Surely you cannot perceive any smell of tobacco about me." "Yes I can," said the professor; "I should not have a chemist's nose if I could not."

There is only one thing in all the catalogue of female beauty that all nations are agreed in admiring, and that is a small mouth. For the rest, white teeth are admired in Europe, and yellow teeth in Asia. We English boast of our rosy maids, while the damsels of Greenland paint their complexions blue or green. We like a straight, or moderately aquiline nose. In Persia the ideal nose is crooked, and in Hayti mothers crush the noses of their children in their infancy in order to make them of the much-admired snub shape. As for the figure, it is well known that plumpness, tending to obesity, is what the Turks and Moors think most of. Rosetti's lady, "dainty thin," would have very few admirers in either Turkey or Africa.

In America there is said to be a growing disinclination to employ women as clerks. It is said that they want more frequent holidays than men, and in general much more attention. No one, not the most enthusiastic advocate of opening all avenues of work to women, will regret that if women are proved unfit for any task it should be taken from them. To give women a preference over men simply because they are women is to do them more harm than good, by teaching them that they may compete in the world's markets on any other terms than that of doing good work. No good ever comes of bolstering up incompetence in either sex. For our own part, we think that women, with their earnest and excitable natures, are less fitted for routine duties, such as those of a clerk, than for work that brings their energy and imagination into play.

The diminution of the population of France has from time to time engaged the attention of politicians, moralists, and sanitarians. Births are few in proportion to the population, and the deaths among young children abnormally large. In the department of the Rhone it is said that children under one year form 48 per cent. of the deaths, in Arèdche as much as 74 per cent. One reason of this enormous infant mortality is that so many French mothers refuse to obey one of Nature's primary laws, and try to bring up their children on artificial food. Without disparaging these very useful substitutes, absolutely essential in many cases, we may say that they should never be resorted to where the natural food can be obtained; and a league has been formed, composed of many distinguished persons, to bring about a better state of public feeling on the subject. The first step has been to bring to Frenchwomen—who are usually, as far as intention goes, loving mothers—a sounder knowledge of physiological laws and of the natural duties of motherhood. Hand-in-hand with this has gone the charitable work of giving to poor mothers those comforts which so often make the difference between strength and weakness to themselves, and consequently to their offspring. We wish the league all success.

